# Five years of experience with the FiLaC™ laser for fistula-in-ano management: long-term follow-up from a single institution

**DOI:** 10.1007/s10151-017-1599-7

**Published:** 2017-03-07

**Authors:** A. Wilhelm, A. Fiebig, M. Krawczak

**Affiliations:** 1 Center of Colorectal and Pelvic Floor Diseases, Aachener Str. 1006-12, 50858 Cologne, Germany; 2 Competence Network of Chronic Venous Diseases, Kiel, Germany; 3 Institute of Medical Informatics and Statistics, Christian-Albrechts-University, Kiel, Germany

**Keywords:** Anal fistula, Repair, Sphincter-preserving, Laser, FiLaC, Faecal incontinence

## Abstract

**Background:**

There are limited data available concerning endofistular therapies for fistula-in-ano, with our group reporting the first preliminary outcomes of the use of the radial fibre Fistula laser Closing (FiLaC ™) device.

**Methods:**

The aim of this study was to assess a cohort of anal fistulae managed with laser ablation plus definitive flap closure of the internal fistula opening over a long-term follow-up. Factors governing primary healing success and secondary healing success (i.e. success after one or two operations) were determined.

**Results:**

The study analysed 117 patients over a median follow-up period of 25.4 months (range 6–60 months) with 13 patients (11.1%) having Crohn’s-related fistulae. No incontinence to solid and liquid stool was reported. Minor incontinence to mucus and gas was observed in two cases (1.7%), and a late abscess treated in one case (0.8%). The primary healing rate was 75/117 (64.1%) overall, and 63.5% for cryptoglandular fistulae versus 69.2% for Crohn’s fistulae, respectively. Of the 42 patients who failed FiLaC™ 31 underwent a second operation (“Re-FiLaC™”, fistulectomy with sphincter reconstruction or fistulotomy). The secondary healing rate, defined as healing of the fistula at the end of the study period, was 103/117 (88.0%) overall and 85.5% for cryptoglandular fistulae versus 92.3% for Crohn’s fistulae. A significantly higher primary success rate was observed for intersphincteric-type fistulae with primary and secondary outcome unaffected by age, gender, presence of Crohn’s disease, number of prior surgeries and the type of flap designed to close the internal fistula opening.

**Conclusions:**

There is a moderate primary success rate using first-up FiLaC™ treatment. If FiLaC™ fails, secondary success with repeat FiLaC™ or other approaches was high. The minimally invasive FiLaC™ approach may therefore represent a sensible first-line treatment option for anal fistula repair.

## Introduction

Recently, alternative therapies have been employed in the management of cryptogenic anal fistula in an attempt to render the surgery more minimally invasive and to reduce the likelihood of post-operative faecal incontinence [1]. These treatments have included ligation of the intersphincteric fistula tract (the LIFT procedure) either with or without the deployment of a biosynthetic mesh [2], or fistula clip closure techniques [3] and a range of endofistular therapies including video-assisted anal fistula treatment (VAAFT) [4], anal fistula plugs [5] and a variety of injected biomaterials [6]. The “Fistula Laser Closing” (FiLaC™) device (Biolitec, Germany) is another endofistular management technique, and its preliminary results in a small cohort were previously reported by our group [7] and two other surgical units [8–10].

The FiLaC™ technique as previously described [7] uses a radial-emitting disposable laser fibre for endofistular therapy and may be supplemented in selected cases with a mucosal advancement anoplasty for control of the internal fistula opening. With other techniques such as bioprosthetic plugs and glues, the principal reasons for fistula recurrence include missed and untreated internal openings, insufficient drainage of the intersphincteric space, missed sidetracks and/or retained remnants of fistula epithelium and granulation tissue [11–13]. The FiLaC™ approach is designed to destroy both the crypt gland and the additional epithelial layer of the fistula track simultaneously by a photothermal effect with coincident obliteration of both the internal and external fistula orifices. This study presents an extension of our original pilot work analysing the first 5 years of our experience employing an unselected approach with the FiLaC™ device in the management of patients with high anal fistulae.

## Materials and methods

The study was approved by the local hospital ethics committee, and all patients undergoing the FiLaC™ procedure provided informed consent. The study analysed 117 patients treated for anal fistulae in a single tertiary referral centre by one experienced colorectal surgeon (AW) between October 2009 and July 2014. Fistulae were classified in accordance with the Parks’ classification system [14], and all patients were preoperatively assessed by clinical examination and proctosigmoidoscopy and classified using three-dimensional (3D) endoanal ultrasonography (B-K Medical, Copenhagen, Denmark) performed by a sonographer experienced in endoluminal anal ultrasound (AW). Very superficial fistulae where fistulotomy could be performed without compromising sphincter function and malignant fistulae were excluded from analysis.

Basic patient demographic data (age, sex) along with fistula type and information (where available) concerning prior surgical treatments were collected. A simple questionnaire was used to assess post-operative continence status. In 110 cases, there was prior drainage of an abscess with removal of side tracks, identification of the internal fistula opening and selective insertion of a seton (where appropriate) into the main fistula track using a 2-mm latex vessel loop (Ethiloop^®^, Ethicon Products, Germany) at the initial operation. Seven patients had a chronic fistula and underwent immediate fistula repair by laser. Before a definitive fistula repair with the FiLaC ™ device, all patients underwent mechanical bowel preparation with 3 L Oralav^®^ (Macrogol, B. Braun Melsungen AG, Germany) and received 2 g intravenous cefuroxime plus 500 mg metronidazole intravenously and two further doses of metronidazole over the first post-operative 24 h.

At the commencement of each treatment, the external and internal orifices of the fistula track were excised, followed by the preparation of a flap. Depending upon the local tissue situation in the area of the internal opening either an advancement, mucosal or anodermal flap was made. The fistula track was cleaned mechanically using a curette and irrigated with saline. The internal opening within the internal sphincter muscle was closed by means of a 2/0 Vicryl© suture, and the laser probe was inserted from the perineal opening. For suprasphincteric type 3 fistulas, the probe was inserted primarily from the internal orifice to reach the “turning point” of the fistula track in order to close the transmuscular fistula component. Then, the internal opening was closed using 2/0 Vicryl©, and the subcutaneous part of the fistula track was treated with the laser from the outer opening as described above.

**Fig. 1:**
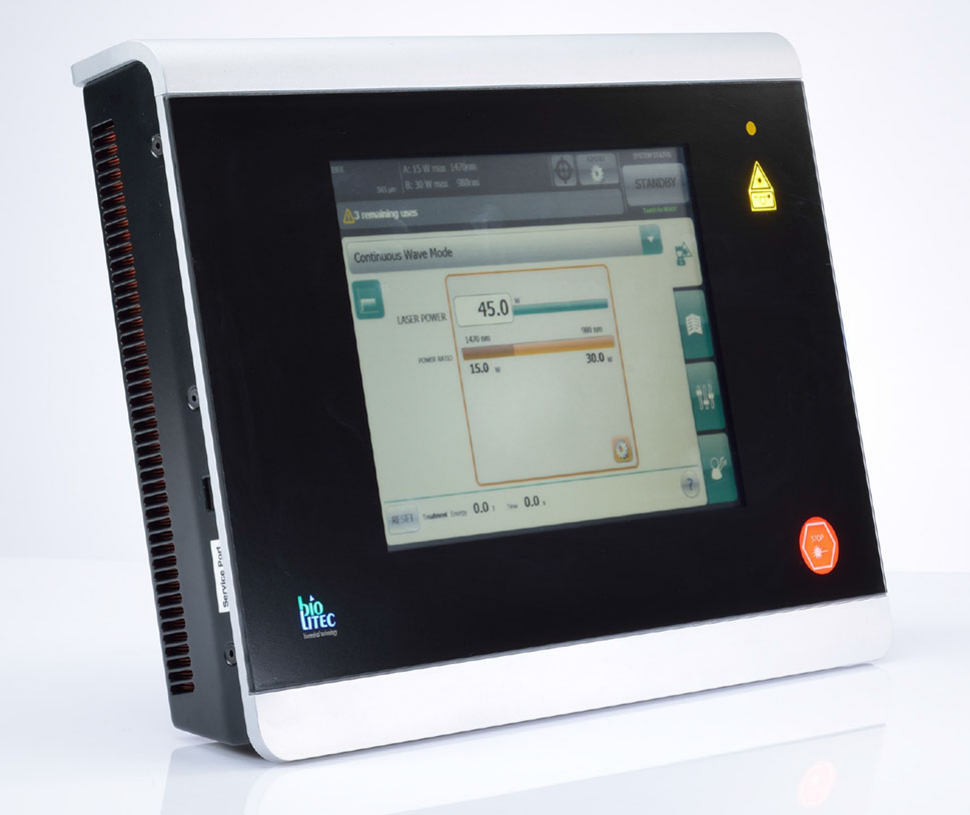
Leonardo DUAL 45© diode laser from Biolitec AG, Germany. Wavelength 980–1470 nm. Maximum energy 15 Watts

**Fig. 2:**
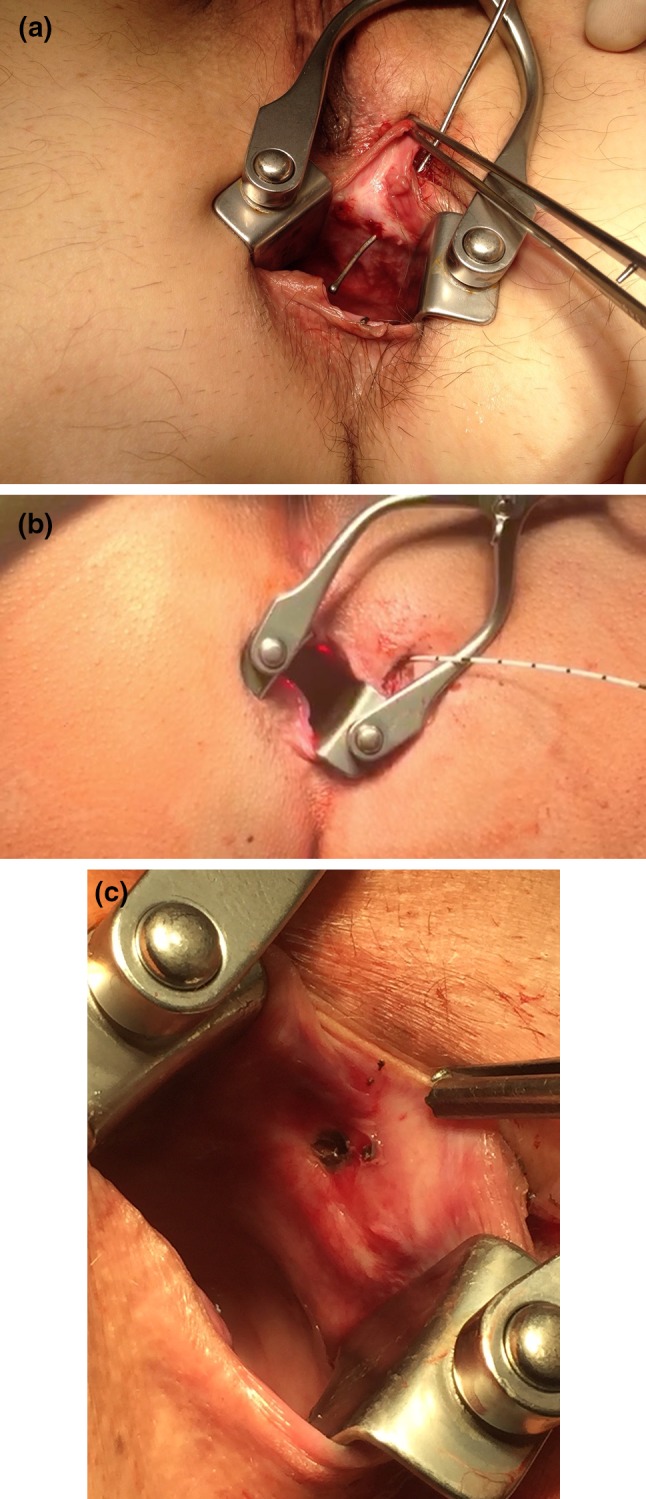
FiLaC™ treatment of a transsphincteric anal fistula. **a** Demonstrating the track with a fistula probe. **b** The laser fibre is inserted into the fistula track. The *red light* indicates the tip of the fibre inside the rectum. **c** Internal orifice following laser treatment. The necrotic tissue will be excised and the defect closed (in this case) with an anodermal flap

The laser probe was inserted through the perineal fistula opening using either a “Ceralas©” (or more recently, a “Leonardo DUAL 45©”) diode laser (Biolitec AG, Germany). This type of laser delivers energy at a wavelength of 1470 nm (Fig. 1) providing an optimal absorption curve in water which is considered to result in a more efficient local tissue shrinkage and protein denaturation. When there is no longer any water in the tissue and the temperature exceeds 100 °C, a white smoke vaporization effect is observed. The use of this wavelength by the radial-tip laser fibre permits destruction of the granulation and epithelial tissue causing a 2- to 3-mm zone of controlled tissue damage with less power (13 W) and a diminished likelihood of perifistular collateral thermal damage [7]. In those suprasphincteric (Parks’ Type 3) fistulae, the laser probe is introduced via the internal fistula opening reaching the “turning point” of the fistula track so as to obliterate the intersphincteric component. For obliteration, the fistula track is treated with a continuous slow retraction of the laser fibre withdrawn at a rate of approximately 1 cm per 3 s (Fig. 2). Treatment progress is assessed by probing the track either with the laser fibre or with a metal fistula probe. Clearly, over-burning or protein denaturation of the treated and surrounding tissue should be avoided. The internal opening was closed using a 2/0 Vicryl© suture creating either a mucosal advancement or an anodermal flap depending upon the local tissue circumstances.

Post-operatively, there were no dietary restrictions, and all patients were placed on stool softeners (Mucofalk^®^) for a 2-week period. Patients were discharged on either the 2nd or the 3rd post-operative day. Follow-up was conducted on the 4th and 10th post-operative days and at 6 weeks, 3 months, 6 months and 1 year thereafter. Further follow-up was carried out at yearly intervals, and patients were instructed to return to the clinic in the interim if symptoms recurred. A fistula was considered to have permanently healed if at 1 year all symptoms had completely disappeared, there was no evidence of recurrence (or persistence) on clinical, proctoscopic and endosonographic examination and there were no additional interventions required. Healing after the first FiLaC ™ laser treatment was defined as primary success, and healing following a repeat operative therapy after initial laser treatment failure was defined as secondary success (Table 1).

**Table 1:** Primary and secondary success rates after FiLaC™

Patient characteristics	*n*	Primary healing				Secondary healing^#^			
		Yes (%)	No (%)	HRR (95% CI)	*p* ^$^	Yes (%)	No (%)	HRR (95% CI)	*p* ^$^
*n*	117	75 (64.1)	42 (35.9)			103 (88.0)	14 (12.0)		
Age^&^		47 (19)	45.5 (19)		0.278	46 (17)	42.5 (20)		0.980
Sex									
Male	82	51 (62.2)	31 (37.8)	***		71 (86.6)	11 (13.4)	***	
Female	35	24 (68.5)	11 (31.5)	1.10 (0.83,1.50)	0.537	32 (91.4)	3 (8.6)	1.06 (0.92,1.21)	0.550
Aetiology									
Cryptoglandular	104	66 (63.5)	38 (36.5)	***		91 (87.5)	13 (12.5)	***	
Crohn	13	9 (69.2)	4 (30.8)	1.09 (0.74,1.61)	0.768	12 (92.3)	1 (7.7)	1.05 (0.89,1.25)	1.000
Park classification									
I	8	8 (100.0)	0 (0.0)	1.63 (1.39,1.93)	0.048	8 (100.0)	0 (0.0)	1.12 (1.05,1.21)	1.000
II	90	55 (61.1)	35 (38.9)	***		80 (88.9)	10 (11.1)	***	
III	13	8 (61.5)	5 (38.5)	1.01 (0.64,1.60)	1.000	10 (76.9)	3 (23.1)	0.87 (0.64,1.18)	0.211
IV	6	4 (66.7)	2 (33.3)	1.09 (0.61,1.97)	1.000	5 (83.3)	1 (16.7)	0.94 (0.65,1.35)	0.528
Closure technique									
MSAF	51	34 (66.7)	17 (33.3)	1.02 (0.77,1.35)	1.000	46 (90.2)	5 (9.8)	1.04 (0.91,1.20)	0.760
Anodermal flap	52	34 (65.4)	18 (34.6)	***		45 (86.5)	7 (13.5)	***	
Mucosal flap	2	1 (50.0)	1 (50.0)	0.76 (0.19,3.10)	1.000	1 (50.0)	1 (50.0)	0.58 (0.14,2.32)	0.277
Suture closure	12	6 (50.0)	6 (50.0)	0.76 (0.42,1.39)	0.341	11 (91.7)	1 (8.3)	1.06 (0.87,1.30)	1.000
Prior fistula repair^§^									
Yes	16	9 (56.3)	7 (43.7)	0.86 (0.55,1.36)	0.577	13 (81.3)	3 (18.7)	0.91 (0.71,1.16)	0.405
No	101	66 (65.3)	35 (34.7)	***		90 (89.1)	11 (10.9)	***	

*HRR* healing rate ratio, *MSAF* mucosal–submucosal advancement flap

^$^ *p* value from Fisher’s exact test except for age, where the *p* value is from a Kruskal–Wallis test

***  Indicates reference group; 9% CI 95% confidence interval

^#^ Defined as healing at the end of study

^§^ Defined as any surgery except I/D of abscess and seton replacement

^&^ Median (interquartile range)

**Fig. 3:**
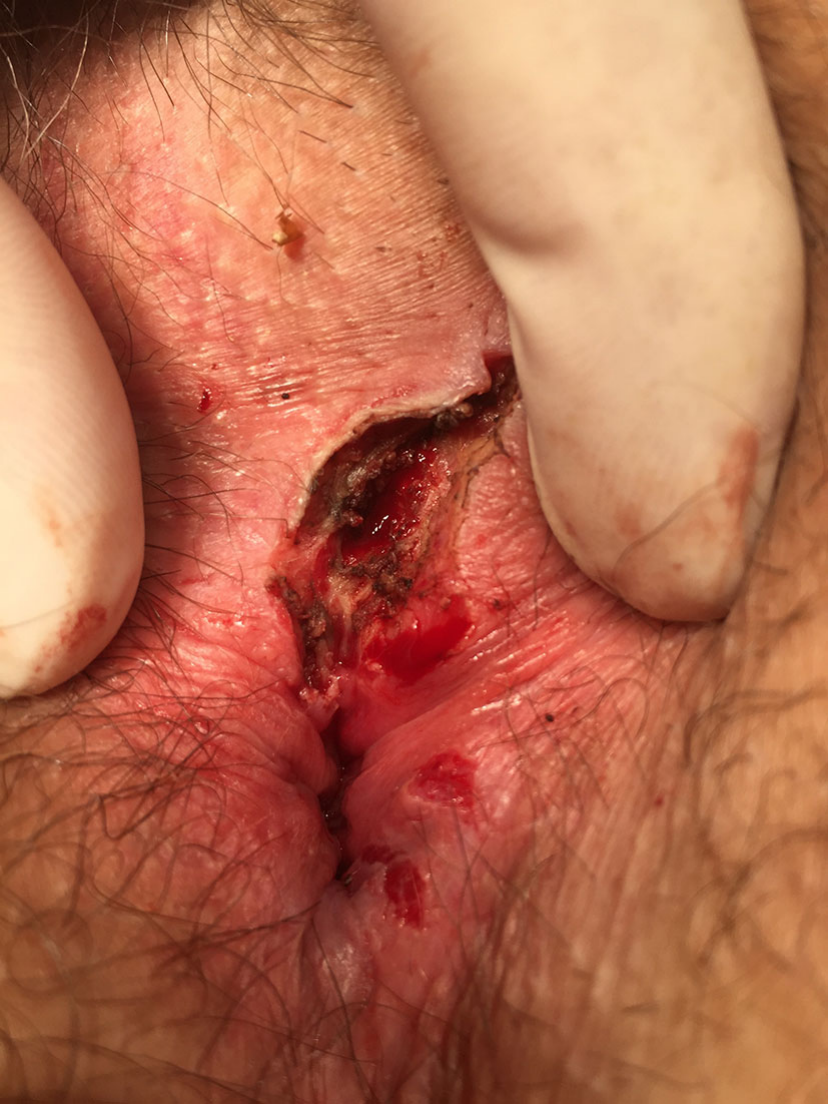
Lay open after failed FiLaC™ treatment. A formerly high transsphincteric fistula became “distalized” (more superficial) permitting delayed fistulectomy without disruption of continence

In the event of treatment failure, there was selective management at the discretion of the surgeon which included repeat laser treatment, fistula excision with partial sphincter reconstruction (if <30% of the sphincter complex was involved) or complete fistula excision with major sphincter reconstruction (if >30% of the sphincter complex was involved), and Fig. 3 shows a lay-open fistulotomy and plug repair (Table 2).

**Table 2:** Secondary success rates after reoperation for an initial failed FiLaC™ procedure

Reoperation	Secondary healing	Secondary success rate (%)
Repeat FiLaC™ procedure	3/5	60
Excision and partial sphincter reconstruction	16/16	100
Excision and major sphincter reconstruction	7/7	100
Gore^®^-Plug	0/1	0
Lay-open fistulotomy	2/2	100

### Statistical analysis

Statistical analysis was performed with SAS 9.4 software (SAS Institute Inc., Cary NC, USA) using the FREQ, UNIVARIATE and NPAR1WAY program procedures, as appropriate. Differences in treatment success between patient subgroups were quantified as healing rate ratios (HRRs) with 95% confidence intervals (95% CI) and assessed for statistical significance using Fisher’s exact test. The largest subgroup was consistently chosen as the reference group. Age distributions were characterized by their median and interquartile range. A Kruskal–Wallis rank test was used to assess age differences between patient subgroups for statistical significance. Two-sided *p* values < 0.05 were considered significant.

## Results

Of the 117 patients, 35 were female and 82 were male (overall median age 46 years; range 17–82 years). The median period of follow-up was 25.4 months (range 6–60 months). In the cohort, 104 fistulae (88.9%) were cryptoglandular in origin and 13 (11.1%) were Crohn’s related. One hundred and thirteen patients (96.6%) had previously undergone surgery including abscess drainage and prior fistula operations. The mean number of operations before FiLaC ™ treatment was 2.4 (±1.7) with a range of 1–9 previous operations. Primary operations had been performed elsewhere in 62 cases (53.0%). Seven patients (6.0%) underwent immediate definitive laser treatment without prior abscess drainage and 11 (9.4%) underwent FiLaC ™ treatment without deployment of a seton following abscess drainage performed elsewhere. A seton was placed in 99 patients (84.6%) with a mean period between seton insertion and definitive fistula treatment of 16.1 (±29.2) weeks. Sixteen patients (13.7%) had a persistent fistula following previous outside fistula repair before laser treatment was performed. Out of this group, 6 (46.0%) had two previous attempts to repair the fistula (Table 3).

**Table 3:** Previous fistula repair before FiLaC™ treatment

	First operation	Second operation
Flap repair	12	2
Plug	4	1
LIFT	0	1

The overall results of the study with primary and secondary success rate in relation to the different variables are listed in Table 1. The primary success rate of the FiLaC™ procedure was 64.1% (75/117) with no difference in this outcome noted between cryptoglandular (63.5%; 66/104) and Crohn’s (69.2%; 9/13) cases (HRR 1.09, 95% CI 0.74,1.61). The secondary success rate was 88.0% (103/117) with no difference noted in this outcome measure between cryptoglandular (85.5%; 89/104) and Crohn’s-related cases (92.3%; 12/13; HRR 1.05, 95% CI: 0.89, 1.29). Sex also lacked a statistically significant effect upon both the primary success rate (male: 62.2%; 51/82 vs. female: 68,5%; 24/35; HRR 1.10, 95 CI 0.83,1.50) and the secondary success rate (86.5%; 71/82 vs. 91.4%; 32/35; HRR 1.06, 95 CI 0.92,1.21). No significant effect on either the primary or secondary success rate was observed for patient age (Kruskal–Wallis test *p* = 0.278 and *p* = 0.980, respectively), the endurance of previous fistula repair (Fisher’s exact test *p* = 0.577 and *p* = 0.405, respectively) or the closure technique of the internal orifice (Fisher’s exact test, all *p* > 0.05). Finally, secondary treatment success rates were not found to be depended on either the number of previous operations or the time interval between the prior and the definitive procedure (data are not shown).

The only statistically significant determinant of treatment success was found to be disease severity. A 1.63-fold increase in primary success rate was observed for fistulae Parks—St. Marks type 1 as compared to type 2 patients, used as the reference group (95% CI 1.39, 1.93). In fact, first treatment was successful in all eight type 1 patients, compared to only 55 of 90 type 2 patients (61%, Fischer’s exact *p* = 0.048). Type 3 and type 4 cases showed similar treatment success rates as type 2 patients, and comparable albeit weaker effects of disease severity were consistently observed for the secondary success rates (Table 1).

No major forms of incontinence (solid, liquid stool or gas) were reported, with minor soiling noted in seven patients (5.9%) (three patients after primary FiLaC™ procedure and four patients after a repeated second fistula surgery). Five of whom had an additional advancement flap to cover the internal opening. Another five of the seven patients responded to rubber band ligation for coincident mucosal prolapse/ectropion. In the total cohort, there was one patient (0.8%) who developed a late abscess and one patient (0.8%) who died of an unrelated cancer during the follow-up period. In those patients where there was initial failure of the FiLaC™ procedure, distalization of the primary track (conversion from a high to a low fistula) was observed in half (21/42) of the cases. At the time of follow-up, 11 patients (9.4%) still have their fistula and have declined a second operative attempt at cure.

## Discussion

Minimally invasive fistula management, which balances clinical effectiveness with an absence of post-operative functional disturbance has been the “holy grail” of anal fistula treatment since Tasci first presented a novel “fistulectome” device in 2003, designed to mechanically core out the primary fistula track [15]. This approach has been extended by Rojanasakul and others with the ligation of the intersphincteric fistula tract (LIFT) procedure [16, 17]. Further extension of this idea towards totally endofistular therapy was advanced by our group’s preliminary report of the FiLaC™ procedure [7] with success in 9/11 cases over a median follow-up of 7.4 months and by Meinero and Mori [3, 18], with their "video-assisted anal fistula treatment” (VAAFT™) technique. These latter two methods were first reported in 2011 in *Techniques in Coloproctology*.

This study reports the long-term outcomes of a cohort of 117 patients presenting with anal fistulae where there was a unit policy towards formal flap closure of the internal fistula opening and as such represents (as far as we are aware) the largest series of FiLaC™—treated anal fistula cases currently available. The primary success rate over a median follow-up of 25.4 months was moderate, and the secondary success rate was high regardless of whether the fistula is cryptoglandular or Crohn’s related in origin. Primary and secondary healing is more likely if the initial fistula is less complicated, and there is no specific advantage of a particular flap technique (when used to close the internal fistula opening definitively). Patient demographics, the number of prior operations or the timing of definitive surgery have no effect on successful outcome. In our study, as in other available reports, no major faecal incontinence was reported [8, 10].

It is recognized that although many sphincter-preserving techniques have provided encouraging preliminary results, over time they have failed to live up to their initial promise [19]. As an outcome measure, a high secondary healing rate is an essential gauge of success of any novel fistula therapy particularly when patients referred to a tertiary institution have had multiple prior failed procedures and where continence may already be partially disturbed. The lack of formal prospective continence assessment is lacking limitation of our study, but it will be a specific focus in future work. Moreover, although the cohort size is comparatively small, it is particularly encouraging that Crohn’s disease does not seem to have any influence on either primary or secondary outcomes following FiLaC™ treatment. The distalization of the primary fistula track in half of the cases where an initial FiLaC™ procedure had failed also permits a much easier repeat fistulectomy with or without the need for a subsequent sphincter repair. In our operative strategy to avoid any kind of faecal incontinence, only two cases of a very low persistent fistula were suitable for a lay-open procedure. In cases with muscular involvement of up to 30% on clinical examination, we did a fistulectomy with “partial” and, in patients with up to 50% involvement, a fistulectomy with a “major” sphincter reconstruction. If a high fistula track persisted and involved more than 50% of the muscle, a second sphincter-saving procedure like “re-FiLaC™” or “Gore^®^-Plug” was performed. The precise mechanism of this shift from a high to a low anal fistula is unclear, but it may be a specific feature of the tissue-modulating effect of the laser.

Lasers have been used as surgical tools for anal diseases for the past four decades [20] with the first report of a CO_2_ laser in 1981 [21] being used to treat three cryptogenic anal fistulae by coring out the fistula track followed by a 2001 Saudi Arabian study reporting six anal fistula cases treated with a KTP solid-state laser combined with fibrin glue instillation [22]. Similar success rates using a CO_2_ laser therapy in complex Crohn’s-related anal fistulae have also been previously reported [23]. In the past, a range of bare-tip diode lasers with variable wavelengths (810, 940 and 980 nm) were used for anal fistulae, borrowing from the effect observed with these types of lasers in a range of endovenous procedures [24]. In the present study, we used a diode laser with a wavelength of 1470 nm and a circular emitting laser fibre, which is estimated to have a 60 times higher efficiency to shrink and seal the fistula track by interacting with water and blood at a lower energy compared to 980 nm. Earlier diode lasers with lower wavelengths (around 980 nm) have been reported to be successful because of their enhanced coagulating effect [8]. The photothermal effect and the optimal shrinkage obtained with a radial-tip fibre result in a more controlled damage, which is confined to the fistula lumen so that the limited radial penetration depth (2–3 mm beyond the fistula track) results in minimal collateral sphincter injury. The reduction in laser energy with the shift from a 980-nm to a 1470-nm probe has also resulted in lower pain scores in a study reported by Giamundo et al. treating 35 patients with the FiLaC™ probe [8].

The use of definitive closure of the internal fistula opening *en passant* with the FiLaC™ procedure remains controversial. Although the flap technique appears to be unassociated with primary or secondary FiLaC™ success rates, our approach has been formally to close the internal fistula orifice and to choose the type of flap closure on a case-by-case basis. It is recognized that formal fistula excision combined with sphincter reconstruction is associated with a 20% rate of post-operative faecal incontinence [25] and that in management of complex fistulas even the traditional flap techniques alone can be associated with incontinence rates of up to 43% [26]. The prospective assessment of the recurrence rate is dependent upon the ability of the laser alone to completely seal the inner part of the fistula track. Giamundo et al. [8, 10] have treated anal fistulae entirely with the laser probe without the need for closure of the internal opening. In this respect, most recurrences appear to occur relatively early following FiLaC™ treatment and are likely the result of fistula reopening with a linking up of the epithelial remnants of small undetected secondary tracks before the denaturation effect of the laser can take effect.

The role of preliminary seton deployment before definitive FiLaC™ therapy requires further examination possibly in a randomized, controlled trial. In our study, the majority of patients had an indwelling seton. In the most recent studies by Giamundo et al. [8, 10], the presence of the seton resulted in a better success rate (although this did not reach statistical significance in a small group of patients). One other study by Özturk et al. [9] reporting high success rates with FiLaC™ therapy did not include seton management as part of a first operative stage. The laser fibre is easier to insert in those patients which already have an indwelling seton, and in this setting the seton can be used to railroad the laser probe across the fistula (Seldinger manoeuvre) and to assist maturation of the principal track by inducing a more homogeneous fibrotic reshaping of the fistula lumen. Such an effect would permit a more uniform shrinkage distribution along the fistula track when the laser is employed in the second-stage procedure. The use of a seton and conversion of a “one-stage” to a “two-stage” procedure will have the benefit of preliminary drainage of abscesses and the intersphincteric space and should encourage small secondary tracks to close improving the likelihood of advancement flap healing if this is used as a supplementary technique.

The FiLaC™ procedure combines the aims of fistula management, namely clinical effectiveness and the maintenance of continence function. Although it is essentially a “blind” procedure that has the potential of missing some small secondary tracks, it is clearly safe and does not induce short-term post-operative sepsis. The long-term results show moderate primary and high secondary healing rates for the majority of fistula types and success rates unaffected by the nature and number of prior procedures or by the presence of Crohn’s disease. The minimally invasive FiLaC™ approach may therefore represent a sensible first-line treatment option for anal fistula repair. Randomized, controlled trials are needed to assess its performance against other minimally invasive fistula therapies.

## References

[1] Limura E, Giordano P (2015). Modern management of anal fistula. World J Gastroenterol.

[2] Han JG, Wang ZJ, Zheng Y (2016). Ligation of Intersphincteric Fistula Tract vs Ligation of the Intersphincteric Fistula Tract plus a bioprosthetic anal fistula plug procedure in patients with transsphincteric anal fistula: early results of a multicenter prospective randomized trial. Ann Surg.

[3] Dubois A, Carrier G, Pereira B (2015). Therapeutic management of complex anal fistulas by installing a nitinol closure clip: study protocol of a multicentric randomised controlled trial–FISCLOSE. BMJ Open.

[4] Meinero P, Mori L (2011). Video-assisted anal fistula treatment (VAAFT): a novel sphincter-saving procedure for treating complex anal fistulas. Tech Coloproctol.

[5] Köckerling F, Alam NN, Narang SK, Daniels IR, Smart NJ (2015). Treatment of fistula-in-ano with fistula plug—a review under special consideration of the technique. Front Surg.

[6] Scoglio D, Walker AS, Fichera A (2014). Biomaterials in the treatment of anal fistula: hope or hype?. Clin Colon Rect Surg.

[7] Wilhelm A (2011). A new technique for sphincter-preserving anal fistula repair using a novel radial emitting laser probe. Tech Coloproctol.

[8] Giamundo P, Geraci M, Tibaldi L, Valente M (2013). Closure of fistula-in-ano with laser—FiLaC™: an effective novel sphincter-saving procedure for complex disease. Colorectal Dis.

[9] Öztürk E, Gülcü B (2014). Laser ablation of fistula Tract: a sphincter-preserving method for treating fistula-in-ano. Dis Colon Rectum.

[10] Giamundo P, Esercizio L, Geraci M, Tibaldi L, Valente M (2015). Fistula-tract laser closure (FiLaC ™): long-term results and new operative strategies. Tech Coloproctol.

[11] Lunnis PJ, Sheffield JP, Talbot IC, Thomson JP, Phillips RK (1995). Persistence of idiopathic anal fistula may be related to epithelialisation. Br J Surg.

[12] Litza EM, van Wijk JJ, Gosselink MP, Doornebosch P, Zimmerman DDE, Schouten WR (2010). Seton drainage prior to transanal advancement flap repair: useful or not?. Int J Colorectal Dis.

[13] Sygut A, Mik M, Trzcinski R, Dziki A (2010). How the location of the internal opening of anal fistulas affect the treatment results of primary transsphincteric fistulas. Langenbecks Arch Surg.

[14] Parks AG, Gordon PH, Hardcastle JD (1976). A classification of fistula-in-ano. Br J Surg.

[15] Tasci I (2003). The fistulectome: a new device for treatment of complex anal fistulas by “Core-Out” fistulectomy. Dis Colon Rectum.

[16] Rojanasakul A, Pattanaarun J, Sahakitrungruang C, Tantiphlachiva K (2007). Total anal sphincter saving technique for fistula-in-ano; the ligation of intersphincteric fistula tract. J Med Assoc Thai.

[17] Zirak-Schmidt S, Perdawood SK (2014). Management of anal fistula by ligation of the intersphincteric fistula tract—a systematic review. Dan Med J.

[18] Meinero P, Mori L, Gasloli G (2014). Video-assisted anal fistula treatment: a new concept of treating anal fistulas. Dis Colon Rectum.

[19] Narang SK, Keogh K, Alam NN, Pathak S, Daniels IR, Smart NJ (2017). A systematic review of new treatments for cryptoglandular fistula in ano. Surgeon.

[20] Iukhvidova ZhM, Makeeva NS, Zinov’eva OI, Sidorova TA (1978). Use of lasers in the treatment of diseases of the anorectal region. Sov Med.

[21] Slutzki S, Abramsohn R, Bogokowsky H (1981). Carbon dioxide laser in the treatment of high anal fistula. Am J Surg.

[22] Salim AS, Ahmed TM (2001). KTP-Laser and fibrin glue for treatment of fistulae in ano. Saudi Med J.

[23] Moy J, Bozdin J (2006). Carbon dioxide laser ablation of perianal fistulas in patients with Crohn’s disease: experience with 27 patients. Am J Surg.

[24] Doganci S, Demirkilic U (2010). Comparison of 980 nm laser and bare-tip fibre with 1470 nm laser and radial fibre in the treatment of great saphenous vein varicosities: a prospective randomized clinical trial. Eur J Vasc Endovasc Surg.

[25] Roig JV, Garcia-Armengol J, Jordan JC, Moro D, Garcia-Granero E, Alonso R (2010). Fistulectomy and sphincteric reconstruction for complex cryptoglandular fistulas. Colorectal Dis.

[26] Göttgens KW, Smeets RR, Stassen LP, Beets G, Breukink SO (2015). Systematic review and meta-analysis of surgical interventions for high cryptoglandular perianal fistula. Int J Colorect Dis.

